# Supplementary material for: Influenza A Virus Infection Reactivates Human Endogenous Retroviruses Associated with Modulation of Antiviral Immunity
Source: Viruses. 2022 Jul 21;14(7):1591. doi: 10.3390/v14071591 (PMC9320126; doi:10.3390/v14071591)
Supplement: Supplementary file 1 [file viruses-14-01591-s001.zip › MotifAnalysis.zip!/MotifOutput_SC35MdNS1/homerResults/motif12.similar.html]

motif12

## Information for motif12

A
G
T
C
A
C
G
T
G
T
A
C
C
G
T
A
C
A
T
G
A
C
T
G
A
C
T
G
A
C
G
T
C
G
A
T
G
T
C
A
C
G
T
A
A
C
G
T
  
Reverse Opposite:  

C
G
T
A
A
C
G
T
A
C
G
T
C
G
T
A
G
T
C
A
G
T
A
C
T
G
A
C
G
A
T
C
A
C
G
T
A
C
T
G
C
G
T
A
T
C
A
G
  

|  |  |
| --- | --- |
| p-value: | 1e-17 |
| log p-value: | -4.088e+01 |
| Information Content per bp: | 1.778 |
| Number of Target Sequences with motif | 8.0 |
| Percentage of Target Sequences with motif | 10.39% |
| Number of Background Sequences with motif | 14.3 |
| Percentage of Background Sequences with motif | 0.03% |
| Average Position of motif in Targets | 99.8 +/- 50.7bp |
| Average Position of motif in Background | 101.0 +/- 45.7bp |
| Strand Bias (log2 ratio + to - strand density) | 3.2 |
| Multiplicity (# of sites on avg that occur together) | 1.25 |
| Motif File: | file (matrix) reverse opposite |

### Similar de novo motifs found

|  |  |  |  |  |  |  |  |
| --- | --- | --- | --- | --- | --- | --- | --- |
| Rank | Match Score | Redundant Motif | P-value | log P-value | % of Targets | % of Background | Motif file |
| 1 | 0.864 | G T A C C G T A A C T G A C G T A C G T A C G T A C G T C G T A G T C A A G C T | 1e-14 | -32.637261 | 11.69% | 0.15% | motif file (matrix) |
